# Assessment of artificial and natural sweeteners present in packaged non-alcoholic beverages (NABs) sold on the Singapore market

**DOI:** 10.1186/s12889-021-11924-0

**Published:** 2021-10-16

**Authors:** Rebecca Tan, Sharon Chew, Xenia Cleanthous, Kimberley Anastasiou, Paige G. Brooker, Theresa Pham, Benjamin P. C. Smith

**Affiliations:** 1 Singapore Institute of Food and Biotechnology Innovation & Innovations in Food & Chemical Safety Programme, Agency for Science, Technology and Research, Singapore, 138671 Singapore; 2 Health & Biosecurity, The Commonwealth Scientific and Industrial Research Organisation, Canberra, ACT 2601 Australia; 3 National Heart Foundation Australia, Docklands, VIC 3008 Australia; 4 Future Ready Food Safety Hub, C/O School of Chemical & Biomedical Engineering, Nanyang Technical University, Singapore, 637459 Singapore

**Keywords:** Market survey, Non-alcoholic beverages, Singapore, Sugar, Sugar substitutes

## Abstract

**Background:**

New Nutri-Grade labelling, aimed at reducing Singaporeans’ sugar consumption will be implemented for all pre-packaged non-alcoholic beverages (NABs) sold in retail outlets from end 2021 onwards. It is expected such labelling will have a major impact on sugar content of beverages, as well as the replacement of sugar with non-caloric alternatives.

**Methods:**

This study used product label data obtained from in-store surveys to investigate sugar and sweetener composition of NABs present on the Singapore market. Using this data we calculated products prospective Nutri-Grade classification in order to compare the current market composition with relation to sugar and/or sweetener use.

**Results:**

Over half of the NABs on market were sweetened with sugar (59%) and were associated with less healthy Nutri-Grades of ‘C’ and ‘D’. The use of natural sweeteners; Stevia and Monk fruit, remains low (6%).

**Conclusion:**

With continuous efforts by the government in promoting public health nutrition, it is expected that there will be a greater usage of sugar substitutes among NABs in response to the upcoming implementation of Nutri-Grade and ever-fluctuating consumers’ demands. The data collected in this study provide a point estimate (July–September 2020) on market composition and use of both sugar and artificial sweeteners in beverages prior to integration of the mandatory labelling requirements.

**Supplementary Information:**

The online version contains supplementary material available at 10.1186/s12889-021-11924-0.

## Background

In recent years, the health consequences of regular consumption of sugar-sweetened beverages (SSBs) have gathered a considerable amount of attention [1–3]. Many studies conducted have documented that the consumption of dietary sugar present in SSBs is directly linked to the increased prevalence of obesity in the population [2–7]. Furthermore, this is positively correlated with increases in the development of metabolic diseases such as type 2 diabetes mellitus (T2DM) [1–4, 8, 9]. SSBs are acknowledged to be one of the main causes of T2DM as it contains the single largest source of sugar in the diet [6, 9].

**Fig. 1:**
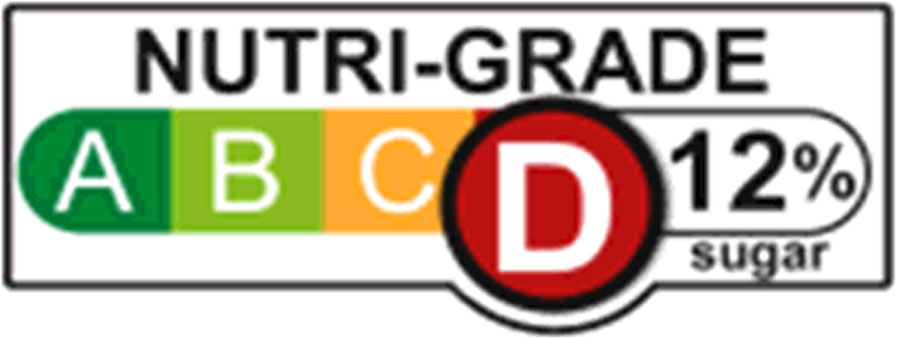
Nutri-Grade Label [10]

According to Singapore’s Health Promotion Board (HPB), the average sugar level for medium- and higher-sugar SSBs per 250 mL serve in Singapore is around 25 g [10]. Beverages containing this amount of sugar represented approximately 50% of the total sales of pre-packaged SSBs in Singapore in 2017 [10]. As part of the ‘War on Diabetes’, the Government will be implementing mandatory front-of-pack nutrition labels, the Nutri-Grade system (Fig. 1), to assist consumers in discerning between healthier and less healthy product options [11, 12]. Such labelling will take effect by the end of 2021 for all pre-packaged and store-made non-alcoholic beverages (NABs) sold in the Singapore market [10].

**Fig. 2:**
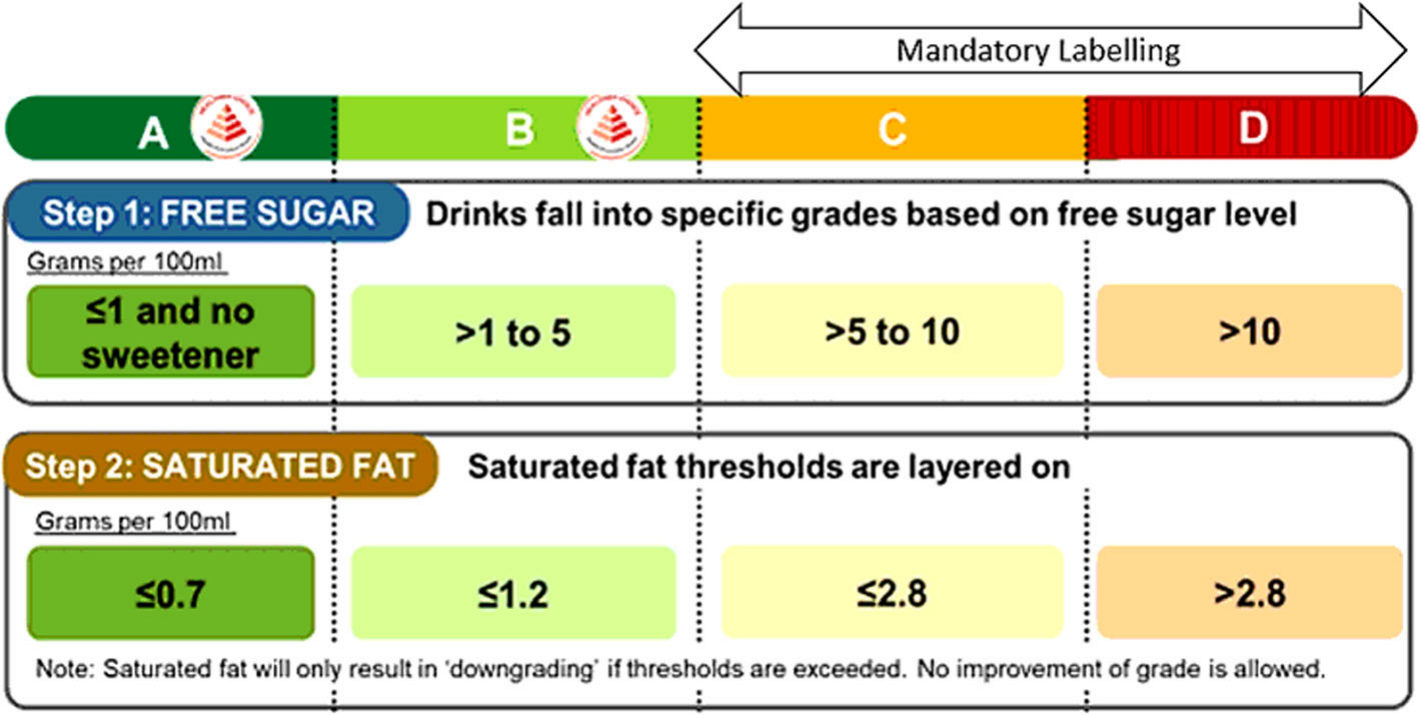
Nutri-Grade Grading System [10]

Under the Nutri-Grade labelling, all NABs will be assigned a summary grade of A-D, based on a set of nutrition thresholds that includes both free sugar, and saturated fat values (Fig. 2) [10]. In addition to the assigned grades, the respective percentages of sugar contained in beverages will be displayed (Fig. 1) [11]. Although such labels are mandatory to be displayed among beverages that fall under grades C and D, it is voluntary for beverages that achieve Grades A and B [10]. It is expected that most beverages labelled with Grades A and B will also qualify for the Healthier Choice Symbol (HCS) label, a classification also awarded by the HPB and is identified by a red pyramid label as displayed in Fig. 2 [10]. HCS requirements, based on nutrient cut-offs set by the HPB, including sugar, saturated fat, trans fat, sodium, calcium and wholegrains, vary for different food groups. Food products are awarded and can display the HCS if they meet those particular nutritional standards [13].

The above labelling requirements are currently restricted to packaged products and are not required for beverages prepared at cafés, restaurants and takeout outlets like Starbucks™ or Boost Juice™. Although store-made drinks such as sugarcane juices, bubble teas and milkshakes, contribute to the overall sugar intake of Singaporeans, menu labelling requirements currently do not exist in Singapore [14]. Up until now, the Singapore government has relied predominantly on public education and awareness to drive behavioural change to reduce sugar intake [15]. The movement towards labelling packaged products is a first step towards managing intake through regulation which can be linked to import compliance and taxation control.

With the implementation of Nutri-Grade, retailers in Singapore are looking towards stocking healthier choices and there is pressure on manufacturers to reformulate beverage formulations. Fully or partially utilizing sugar substitute(s) is part of the approach to meet this goal [16, 17]. Table 1 provides a summary of the available sugar substitutes in the market, categorized into its respective classes based on nomenclature; caloric contribution and source of origin [18, 19].

**Table 1:** Classification and Types of Sugar Substitutes Available in products sold in Singapore [18–23]

Classification of Sugar Substitutes	Types of Sugar Substitute(s) Available	Sweetness Intensity relative to Table Sugar
Caloric/Low-caloric Artificial Sweeteners	Aspartame	200 times sweeter
Natural Sweeteners	Steviol Glycosides	200–400 times sweeter
	Monk Fruit Extract	100–250 times sweeter
Sugar Alcohols (Sweetness Intensity of Sucrose valued at 1.0)		Sweetness Intensity relative to Sucrose:
	Erythritol	0.812
	Lactitol	0.4
	Maltitol	0.9
	Sorbitol	0.6
	Xylitol	1.0
Zero-Calorie Artificial Sweeteners	Acesulfame Potassium (Acesulfame-K)	200 times sweeter
	Saccharin	200–700 times sweeter
	Sucralose	600 times sweeter

Evidence investigating the health effects of consuming beverages containing sugar substitute(s) is conflicting and inconclusive [16, 17, 24–28]. This is in part due to a lack of solid knowledge of the product compositions on market in relation to the proportion of sugar substitutes and their contribution to the diet. Some studies have shown that regular consumption of artificially sweetened beverages was associated with increased health risks, including obesity, cardiovascular diseases, and metabolic syndrome [24–26]. Meanwhile, other studies indicated that regular consumption of artificially sweetened beverages had little to no effect on weight gain and even promoted weight loss [27–30].

Due to the differences between studies, such as study duration and design, it is difficult to ascertain the strength and direction of the relationship between the consumption of artificially and/or naturally sweetened beverages and cardiometabolic health over prolonged periods of time and how this compares with sugar [31]. Despite differences in research outcomes about the effects of artificially and/or naturally sweetened beverages on public health, replacing free sugars with sugar substitutes will undoubtedly reduce the total dietary energy intake [28–30] and increased use in the use of sweeteners in products marketed in Singapore is an expected outcome of the implementation of the Nutri-Grade system. Furthermore, with the growing popularity of purchasing clean label and natural products; where the term ‘clean labels’ is commonly used to describe products lacking artificial flavours, colours, and preservatives, whose ingredient lists are simple with no unpronounceable or ‘chemical sounding’ additives, the promotion and/or usage of natural and organic sweeteners is predicted to rise [32, 33].

The objective of this study was to obtain baseline data, and position ourselves to monitor future product trends in sugar and sweetener use in non-alcoholic beverages. This study uses package labelling data obtained from in-store surveys to investigate sugar and sweetener composition of NABs present on the Singapore market, as well as assess their future Nutri-Grade classification. The data collected in this study provide a point estimate (July–September 2020) on market composition and use of both sugar and artificial sweeteners in beverages prior to integration of the mandatory labelling requirements.

## Methods

### Data source

All relevant information on the beverages packaging such like nutritional information panel (NIP), ingredients list and various product descriptor information (e.g., health and nutrition claims, country of manufacture and origin, products that needed to be reconstituted with water or milk before consumption) was collected physically in stores using FoodTrack™, an Australian food-based composition data collection platform and database technology [34]. Trained personnel visited a total of 12 store locations in Singapore across the four major retail supermarkets: Cold Storage, Giant, NTUC FairPrice and Sheng Siong, and two major convenience stores: 7-Eleven and Cheers after obtaining permission from the respective store managers. Based on an assessment of market share and product listings, it was estimated that these stores covered greater than 90% of the beverages sold on the Singapore market. On-package information on all beverages positioned in all areas/sections of supermarkets and convenience stores that are categorised as NABs were collected. Data acquisition occurred between July and September 2020.

### Study sample

In this study, NABs were grouped into twelve sub-categories based on their ingredient and nutrient compositions. This grouping was based around Australian categories of Sugar-Sweetened Beverages or SSBs in order to align with the current FoodTrack™ database categorisation system. Sub-categories included: (1) Asian/Asian-style drinks; (2) carbonates; (3) coconut water; (4) cordials, syrups, concentrates; (5) energy drinks; (6) flavoured drinks; (7) kombucha; (8) *milk-based/iced coffees; (9) *milk-based/iced teas; (10) ready-to-drink (RTD) vinegars; (11) sports/isotonic drinks; and (12) vitamin drinks (see Appendix). Products excluded from data collection and analysis included drinking yoghurt, flavoured milk and milk alternatives, juices (fruits and vegetables), plain bottled water, and milk-based beverages (*milk-based iced coffees and teas where milk was added as an ingredient, not as the basis of the product/characterising ingredient). Although these excluded beverages may contain added sugar, these products are not categorised as NABs/SSBs in FoodTrack™ and consequently have not been included in the analysis for this paper.

### Data collection

Photographs of the entire beverage packaging were captured, and relevant qualitative and quantitative information was manually transcribed and entered into the FoodTrack™ App for each beverage sampled before being uploaded into a remote database. The following fields of information were extracted: ingredients list, instructions usage, HCS logos and respective claims) NIP values, serving size and net weight/volume. All values and calculations reported on the NIP were normalised to per 100 g or per 100 mL.

All beverages considered NABs/SSBs, based on our category inclusion criteria, were sampled in each store visited. To ensure no products were missed, collectors were instructed to thoroughly check all aisles in each store, not only those aisles where beverages were primarily sold. The thoroughness of sample collection was verified by repeat visits using different collectors.

### Data analysis

Data was analysed using Microsoft Excel™ and Excel Stats™ tools (Microsoft Office 2012 – Version 16.0.13530.20368). Beverages were classified according to their sub-categories listed in the inclusion criteria and different parameters were applied to compare the results, including determining the average energy, carbohydrate, sugar and saturated fat contents across sub-categories, identifying the types and sum of primary taglines labelled on beverages that provide further product differentiation based on the amount of added sugar content, and comparing the types of sugar and sugar substitutes used across all beverages. Based on the types of sugar and/or sugar substitutes used, each beverage was classified according to one of its pre-defined sub-categories; unsweetened, intensely sweetened, calorically sweetened and both calorically and intensely sweetened beverages (where ‘calorically sweetened’ refers to all beverages that still use sugar in their formulations, regardless of whether or not they use additional artificial sweeteners) [35]. The number of sugar substitutes used in each beverage was tabulated and categorised based on Table 1 [18–22].

Descriptive statistics were presented (mean, standard deviation, ranges, and percentages) for the distinct categories and data points, and subsequently combined for overall analysis across the twelve overarching beverage sub-categories. Nutrient (g) data were rounded to one decimal place, and energy (kJ) data were rounded to the nearest whole number.

### Nutri-grade assessment

Based on the NIP values, beverages were further re-categorised into their respective nutrient summary labels in accordance with the Nutri-Grade Grading System guidelines in Fig. 2 [10]. The proportion of beverages’ nutritional contents that met the various nutrient claims and summary grades of A to D were summarised and presented as percentages. The average total sugar content was also expressed as a percentage of the total beverage volume.

## Results

In total, 883 NABs were collected and assessed in this study. Thirty-five beverages were excluded from the NIP and Nutri-Grade data analyses as their product labels did not carry any nutritional data and thus the presence or absence of sugars and/or sweeteners could not be ascertained. The absence of such data is not surprising, as under the Singapore food legislation, NIPs are only required for pre-packed foods that carry nutrition and health claims, edible fats and oils, and special purpose food. Most products nonetheless contained NIPs, as evidenced by our data, which is likely more so a reflection of other country’s labelling requirements due to the high number of imported products entering Singapore. Of the remaining 848 beverages assessed, on average, vitamin drinks contained the highest sugar content of 22.5 g per 100 mL/g across all sub-categories. This was also reflected in their corresponding high energy (390 kJ, 93kCal) and total carbohydrate values (23.4 g). Conversely, the saturated fat content of beverages ranged from 0.0 to 0.7 g as it was observed that saturated fat contents were mainly influenced by the presence of animal-based ingredients such as milk and milk derivates in several tea and coffee beverages. NABs with the highest saturated fat contents (0.7 g per 100 mL/g) still fell within the lowest threshold (Grade A) for saturated fat limits, therefore, all NABs were graded based on their sugar content. Their grades remained unchanged after taking their respective saturated fat contents into account (Table 2).

**Table 2:** Nutritional Information of Non-Alcoholic Beverages Across Sub-Category per 100 mL/g (*n* = 848)

	n	Mean (SD)			
		Energy (kJ/kCal)	Carbohydrates (g)	Sugar (g)	Saturated Fat (g)
Asian/Asian-style Drinks	118	125/30 (44)	7.2 (2.2)	6.8 (2.1)	0.1 (0.2)
Carbonates	219	109/26 (79)	6.3 (4.0)	6.0 (3.9)	0.2 (0.4)
Coconut Water	36	94/22 (24)	5.3 (0.8)	4.5(0.8)	0.1 (0.4)
Cordials, Syrups, Concentrates	33	194/46 (210)	11.3 (12.4)	11.1 (12.8)	0.0 (0.0)
Energy Drinks	30	164/39 (109)	9.2 (6.9)	7.3 (6.7)	0.0 (0.0)
Flavoured Drinks	40	177/42 (52)	10.2 (3.0)	9.0 (3.0)	0.2 (0.4)
Kombucha	18	61/15 (22)	3.6 (1.5)	3.0 (1.0)	0.1 (0.2)
Milk Based/Iced Coffees	6	199/47 (29)	8.5 (1.2)	6.7 (1.2)	0.7 (0.2)
Milk Based/Iced Teas	242	155/37 (303)	7.8 (12.4)	5.1 (3.4)	0.1 (0.3)
RTD Vinegars	41	374/89 (318)	21.8 (19.1)	19.6 (17.2)	0.0 (0.0)
Sports/Isotonic Drinks	54	119/28 (214)	7.0 (12.4)	6.8 (11.9)	0.0 (0.0)
Vitamin Drinks	11	390 (582)	23.4 (35.3)	22.5 (33.8)	0.0 (0.0)
Overall	848	149 (215)	8.3 (10.6)	6.9 (7.9)	0.1 (0.3)

**Fig. 3:**
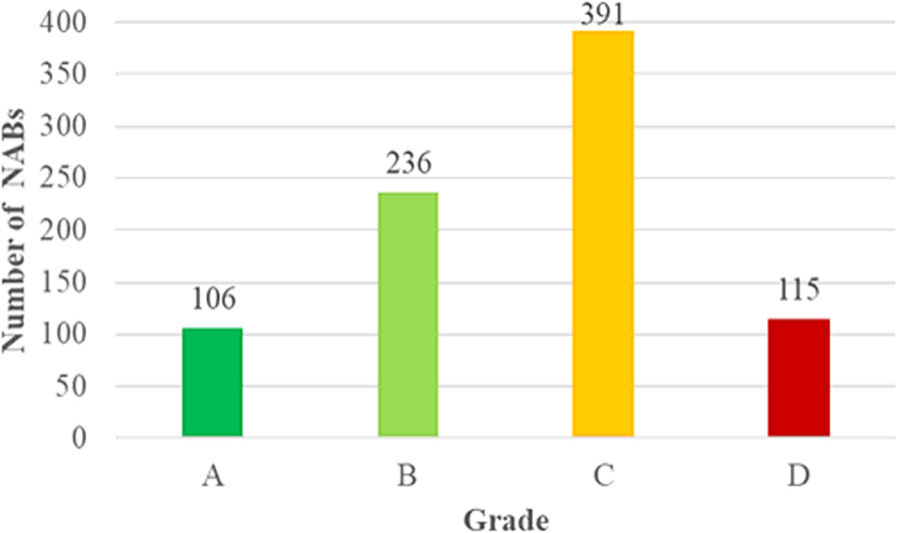
Proportion of Non-Alcoholic Beverages in each Nutri-Grade (*n* = 848)

Majority of NABs collected fall under Grade C (*n* = 391, 46%) of the Nutri-Grade, and more than half (*n* = 506, 60%) of the beverages sold on the market fall into the unhealthy bands either Grades C or D (Fig. 3). These beverages would be required to adopt the mandatory labelling requirement if the Nutri-Grade policy was implemented. The average sugar content across all NABs is 6.9 g per 100 mL/g (average range of 3.0 to 22.5 g) and would be predominantly assigned to a yellow label; Grade C (Table 3).

**Table 3:** Proportion of Sub-categories of Non-Alcoholic Beverages Classified in each Nutri-Grade (*n =* 848)

Free Sugar Thresholds Level (g per 100 mL/g)	n	n (%)				Ave. Nutri-Grade
		Grade A (≤1 and no sweetener)	Grade B (> 1 to 5)	Grade C (> 5 to 10)	Grade D (> 10)	
Asian/Asian-style Drinks	118	4 (3)	13 (11)	97 (82)	4 (3)	C
Carbonates	219	22 (10)	83 (38)	74 (34)	40 (18)	C
Coconut Water	36	–	30 (83)	6 (17)	–	B
Cordials, Syrups, Concentrates	33	2 (6)	2 (6)	16 (49)	13 (39)	D
Energy Drinks	30	4 (13)	11 (37)	5 (17)	10 (33)	C
Flavoured Drinks	40	4 (10)	6 (15)	10 (25)	20 (50)	C
Kombucha	18	–	18 (100)	–	–	B
Milk Based/Iced Coffees	6	–	1 (17)	5 (83)	–	C
Milk Based/Iced Teas	242	63 (26)	49 (20)	122 (50)	8 (3)	C
RTD Vinegars	41	7 (17)	12 (29)	7 (17)	15 (37)	D
Sports/Isotonic Drinks	54	–	7 (13)	46 (85)	1 (2)	C
Vitamin Drinks	11	–	4 (36)	3 (27)	4 (36)	D
Overall	848	106 (13)	236 (28)	391 (46)	115 (14)	C

Nearly half (41%) of the NABs were compliant with the national nutritional guidelines and were thus eligible for the display of various comparative HCS logos and their accompanying taglines (Table 4). The HCS logo was absent on product labels of four out of 12 beverage sub-categories (i.e., energy drinks, kombucha, RTD vinegars and cordials, syrups, concentrates). Close to one third of NABS carried the ‘Lower in Sugar’ HCS tagline (*n* = 268, 30%), followed by ‘Sugar Free’ (*n* = 52, 6%) and ‘No Added Sugar’ (*n* = 41, 5%) taglines. Milk-based/iced teas were observed to have the highest count of each tagline statement.

**Table 4:** Proportion of Non-Alcoholic Beverages Carrying HCS and Its Accompanying Taglines (*n* = 883)

	n	n (%)			
		Lower in Sugar	No Added Sugar	Sugar Free	No Tagline
Asian/Asian-style Drinks	124	54 (44)	–	1 (1)	69 (56)
Carbonates	222	55 (25)	1 (1)	23 (10)	143 (64)
Coconut Water	38	12 (32)	18 (47)	–	8 (21)
Cordials, Syrups, Concentrates	48	–	–	–	48 (100)
Energy Drinks	30	–	–	–	30 (100)
Flavoured Drinks	41	9 (22)	2 (5)	–	30 (73)
Kombucha	18	–	–	–	18 (100)
Milk Based/Iced Coffees	6	1 (17)	–	–	5 (83)
Milk Based/Iced Teas	249	89 (36)	20 (8)	23 (9)	117 (47)
RTD Vinegars	42	–	–	–	42 (100)
Sports/Isotonic Drinks	54	43 (80)	–	5 (9)	6 (11)
Vitamin Drinks	11	5 (46)	–	–	6 (55)
Overall	883	268 (30)	41 (5)	52 (6)	522 (59)

Slightly more than one quarter of NABs (*n* = 241, 27%) contain the use of sugar substitute(s) in their product formulations. The usage of sugar substitutes was found to be most popular among energy drinks (*n* = 24, 80%), but completely absent among vitamin drink products. Instead, all vitamin drinks were all calorically sweetened (*n* = 11, 100%). Within each of the twelve sub-categories, there is still a small proportion of beverages that are calorically sweetened with sugar. The prevalence of NABs classified as calorically sweetened beverages remains the highest at 59% (Table 5).

**Table 5:** Frequency of Sugar Substitutes Used and Its Corresponding Classification (*n* = 883)

	n	n (%)					
		Contain the Use of Sugar Substitute(s)			Do Not Contain the Use of Sugar Substitute(s)		
		Intensely Sweetened Beverages	Both Calorically and Intensely Sweetened Beverages	Total	Unsweetened Beverages	Calorically Sweetened Beverages	Total
Asian/Asian-style Drinks	124	–	27 (22)	27 (22)	7 (6)	90 (73)	97(79)
Carbonates	222	28 (13)	70 (32)	98 (45)	26 (12)	98 (44)	124 (56)
Coconut Water	38	–	6 (16)	6 (16)	27 (71)	5 (13)	32 (84)
Cordials, Syrups, Concentrates	48	2 (4)	–	2 (4)	–	46 (96)	46 (96)
Energy Drinks	30	9 (30)	15 (50)	24 (80)	–	6 (20)	6 (20)
Flavoured Drinks	41	3 (7)	8 (20)	11 (27)	2 (5)	28 (68)	30 (73)
Kombucha	18	–	1 (6)	1 (6)	–	17 (94)	17 (94)
Milk Based/Iced Coffees	6	–	2 (33)	2 (33)	–	4 (67)	4 (67)
Milk Based/Iced Teas	249	2 (1)	48 (19)	50 (20)	52 (21)	147 (59)	199 (80)
RTD Vinegars	42	7 (17)	6 (14)	13 (31)	10 (24)	19 (45)	29 (69)
Sports/Isotonic Drinks	54	5 (9)	2 (4)	7 (13)	–	47 (87)	47 (87)
Vitamin Drinks	11	–	–	–	–	11 (100)	11 (100)
Overall	883	56 (6)	185 (21)	241 (27)	124 (14)	518 (59)	642 (73)

* Both calorically and intensely sweetened beverages contain both added sugar and one or more sugar substitute(s)

Based on the four types of sugar substitutes (classified in Table 1**)**, zero-calorie artificial sweeteners were the most commonly used (*n* = 264, 30%), followed by sugar alcohols (*n* = 63, 7%), natural sweeteners (*n* = 55, 6%), and lastly, caloric artificial sweeteners (*n* = 17, 2%). The presence of sucralose (*n* = 143, 16%) and acesulfame-k (*n* = 120, 14%) were comparable in terms of its frequency and prevalence used among NABs containing zero-calorie artificial sweeteners. Both substitutes were predominantly used in carbonated drinks. Carbonated drinks also contained the most diverse range of sugar substitutes (7 different types). The next most popular alternative to zero-calorie artificial sweeteners were sugar alcohols (*n* = 63, 7%), with sorbitol being the most utilized sugar substitute (*n* = 28, 3%). Among the natural sweeteners used in NABs, a higher percentage of steviol glycosides (*n* = 47, 5%) was used as compared to monk fruit extract (*n* = 8, 1%). Although aspartame, the only caloric artificial sweetener, was the least popular sugar substitute (*n* = 17, 2%), it was mainly found in carbonated beverages (*n* = 16, 7%) (Table 6). The aforementioned then gives relevance to Table 7, presenting a combination of the total number and classification of sweeteners used across in each sub-category, where most beverages contained a maximum of two different types of sugar substitutes and classifications.

**Table 6:** Types and Frequency of Sugar Substitutes Used in Non-Alcoholic Beverages (*n* = 882)

	n	n (%)												
		Caloric Artificial Sweetener	Natural Sweeteners			Sugar Alcohols					Zero-Calorie Artificial Sweeteners			
		Aspartame	Monk Fruit Extract	Steviol Glycosides	Total	Erythritol	Inositol	Maltitol	Sorbitol	Total	Acesulfame-K	Saccharin	Sucralose	Total
Asian/Asian-style Drinks	124	–	4 (3)	10 (8)	14 (11)	–	–	–	12 (10)	12 (10)	1 (1)	–	4 (3)	5 (4)
Carbonates	221	16 (7)	–	9 (4)	9 (4)	7 (3)	–	–	4 (2)	11 (5)	82 (37)	1 (1)	83 (38)	166 (76)
Coconut Water	38	–	–	–	–	–	–	–	–	–	–	–	6 (16)	6 (16)
Cordials, Syrups, Concentrates	48	–	2 (4)	2 (4)	4 (8)	–	–	–	–	–	–	–	–	–
Energy Drinks	30	1 (3)	–	–	–	1 (3)	17 (57)	2 (7)	–	20 (67)	7 (23)	–	14 (47)	21 (70)
Flavoured Drinks	41	–	–	2 (5)	2 (5)	–	–	5 (12)	–	5 (12)	–	–	4 (10)	4 (10)
Kombucha	18	–	–	1 (6)	1 (6)	1 (6)	–	–	–	1 (6)	–	–	–	–
Milk Based/Iced Coffees	6	–	–	–	–	–	–	–	–	–	–	–	2 (33)	2 (33)
Milk Based/Iced Teas	249	–	–	14 (6)	14 (6)	2 (1)	–	–	9 (4)	11 (5)	24 (10)	–	22 (9)	46 (19)
RTD Vinegars	42	–	–	9 (21)	9 (21)	–	–	–	3 (7)	3 (7)	1 (2)	–	3 (7)	4 (9)
Sports/Isotonic Drinks	54	–	2 (4)	–	2 (4)	–	–	–	–	–	5 (9)	–	5 (9)	10 (18)
Vitamin Drinks	11	–	–	–	–	–	–	–	–	–	–	–	–	–
Overall	882	17 (2)	8 (1)	47 (5)	55 (6)	11 (1)	17 (2)	7 (1)	28 (3)	63 (7)	120 (14)	1 (0)	143 (16)	264 (30)

* 1 product, Le Le China Apple Flavoured Beverage (Carbonates), was excluded from this analysis due to insufficient label information

**Table 7:** Total Average Number and Classification of Sugar Substitutes(s) Used Across each Sub-Category (*n* = 883)

	n	n (%)									
		Number of Sugar Substitute(s) Used						Number of Classification of Sugar Substitute(s) Used			
		0	1	2	3	4	Not Otherwise Defined	0	1	2	Not Otherwise Defined
Asian/Asian-style Drinks	124	97 (78)	23 (19)	4 (3)	–	–	–	97 (78)	23 (19)	4 (3)	–
Carbonates	222	124 (56)	5 (2)	79 (36)	13 (6)	–	1 (1)	124 (56)	71 (32)	26 (12)	1 (1)
Coconut Water	38	32 (84)	6 (16)	–	–	–	–	32 (84)	6 (16)	–	–
Cordials, Syrups, Concentrates	48	46 (96)	–	2 (4)	–	–	–	46 (96)	2 (4)	–	–
Energy Drinks	30	6 (20)	13 (43)	7 (23)	1 (3)	3 (10)	–	6 (20)	15 (50)	9 (30)	–
Flavoured Drinks	41	30 (73)	11 (27)	–	–	–	–	30 (73)	11 (27)	–	–
Kombucha	18	17 (94)	–	1 (6)	–	–	–	17 (94)	–	1 (6)	–
Milk Based/Iced Coffees	6	4 (67)	2 (33)	–	–	–	–	4 (67)	2 (33)	–	–
Milk Based/Iced Teas	249	199 (80)	29 (12)	21 (8)	–	–	–	199 (80)	50 (20)	–	–
RTD Vinegars	42	29 (69)	10 (24)	3 (7)	–	–	–	29 (69)	11 (26)	2 (5)	–
Sports/Isotonic Drinks	54	47 (87)	2 (4)	5 (9)	–	–	–	47 (87)	7 (13)	–	–
Vitamin Drinks	11	11 (100)	–	–	–	–	–	11 (100)	–	–	–
Overall	883	642 (73)	101 (11)	122 (14)	14 (2)	3 (0)	1 (0)	642 (73)	198 (22)	42 (5)	1 (0)

*Not Otherwise Defined: (*n* = 1): Data from Le Le China Apple Flavoured Beverage (Carbonates) as types of sweeteners used were not specified on product label

## Discussion

Among the 883 unique NABs identified in this study, the majority of beverages (59%) were calorically sweetened, 14% were unsweetened and the remaining 27% were sweetened with the use of sugar substitutes. Results from this study align with findings from other studies, indicating that SSBs make up a considerable source of calories (sugar) in the diet [9, 10, 34]. Over half of Singaporeans’ total daily sugar intake is derived from pre-packaged SSBs (64%), according to a national nutrition survey conducted by the HPB in 2018 [9–11]. Thus, the uptake and promotion of artificial sweeteners is an opportunity for consumers to reduce their overall caloric intake from SSBs. Coupled with the introduction of Nutri-Grade, this study provides a point of discussion for future research investigating the use and uptake of sugar substitutes into the market, including new and novel forms of sweeteners such as Monk fruit [35, 36].

Carbonated drinks as a product sub-category had the most diverse use of sugar substitutes; 7 unique types as well as multiple use of different sweeteners in the same product (Table 7). These findings are consistent with a recent study, which assessed the presence of sugar substitutes used in twelve major food and beverage subgroups across four countries [37, 38]. It was reported that Chile encompassed the highest magnitude of products containing non-nutritive sweeteners in most categories along with soft drinks/sodas dominating the total amount of non-nutritive sweeteners being used within the beverage’s category [37, 38]. Similarly, the top three sugar substitutes used in carbonated drinks; sucralose, acesulfame-K and aspartame, were in line with findings from a study conducted by Buffini et al., in investigating the most widely utilised and consumed sweeteners present among the Irish adult population diet [39].

Conversely, vitamin drinks did not contain any sugar substitutes, but contained the highest energy, carbohydrate, and sugar values across all twelve sub-categories. These high values are reflected in the Nutri-Grade assigned to these beverages – ‘red’ Grade D label for highest sugar levels (23%). Examples of vitamin drinks include brands such as Glacéau and YOU-C1000. More often than not, the major selling point of vitamin drinks is their promotion of functional and hydration benefits associated with achieving optimal sports/athletic and health performance from the fortification of vitamins, minerals, and electrolytes [40, 41]. Thus, the replacement of natural sugars with sugar substitutes may not be a key manufacturing consideration in product development or reformulations in appealing to consumers. That said, there is still a fraction of consumers who instinctively view and associate such beverages incorrectly with healthier alternatives, which could be a cause of concern if these drinks are consumed frequently and/or in the absence of physical activity.

More than half of NABs in this study have high sugar contents and would be assigned either ‘yellow’ Grade Cs (46%) or ‘red’ Grade Ds (14%), paralleling an analysis conducted by HPB that found that the proportion of beverages classified as Grade C was approximately 50% and accounted for 51% of the total sales in the current market [10–12]. As Nutri-Grade is gradually implemented, it will be worthwhile to track how the usage of both sugar and sugar substitutes in NABs may vary in the short and long-term. Such findings are reflected in other studies of countries which introduced similar nutrient labelling systems, prompting the need to undertake product reformulations to improve the overall healthfulness of food retail markets [42–45]. One such example demonstrated was the Health Star Rating (HSR) programme implemented in New Zealand [45]. Since the implementation of the HSR programme, improvements were observed in certain nutritional compositions of packaged food products particularly, energy, fibre, and sodium density [45].

Another strong example is the impact of Chile front-of-pack labelling to warn consumers about the health risks of sugar over-consumption [43, 44]. Chile became the first country to administer mandatory warning labels on the front of packs for sugar levels in food in 2016 [43, 44]. As a result, the pressure to maintain consumer loyalty and product sales led many food companies to reformulate their products by replacing sucrose with non-caloric sweeteners, thereby supporting the government initiative in decreasing the average overall energy intake of consumers, and effectively demonstrating the significant downstream effects labelling initiatives can have on shifting consumer purchase behaviour [43, 44].

The similarity in background rationale and objectives of the New Zealand (NZ) and Chilean policies with Singapore’s Nutri-Grade suggests that, upon implementation, the reduction in the proportion of sugar in NABs is attainable. Consequently, it is hypothesised that for future new beverage development or reformulations, there will be a shift away from added sugar in NABs towards sugar substitutes. This assists the beverage industries in leveraging off the anticipated/forecasted increased consumer demand for NABs in qualifying the desirable healthy Nutri-Grade label and/or HCS logos and its respective tagline(s) [12, 13]. It will also be interesting to track how the new labelling is understood and perceived by consumers. Both the HCS and Nutri-Grade labelling are marketed collectively on Grades A and B beverage packaging so it will be interesting to observe which categorisation drives future consumer choice [12–15]. For instance, will consumers differentiate between a Grade A beverage that contains zero added sugar versus 1% added sugar or just equate all Grade A beverages as equivalent? Equally, is a Grade B beverage with 1% sugar but the addition of sweeteners perceived differently? [12–15]. At this stage, we cannot make any informed comment, but it is something that will be interesting to track in the future and the methodology described in this study provides a tool for us to do so.

An expansion of the usage of more sugar substitutes by formulators to ensure an A or B grading is a tangible prospective next step after the implementation of the Nutri-Grade system [46–48]. What impact this has on the diet and health outcomes of Singaporeans cannot be ascertained by this study nor is it the intention of this paper to debate the health benefits nor implications of sweeteners versus sugar, however, monitoring such trends is important. Some concerns have been raised over the use of artificial sweeteners in foods on top of the growing concerns many have with sugar [49–52]. Thus, a proper understanding and assessment of the change in trend of sweeteners and sugar use in the Singapore diet, alongside measures of population health, is important so that we can identify emerging correlations between product composition and changing dietary practices and health metrics as well as adequately assess the level of impact of any new data on the safety of sweeteners present in the Singapore diet.

We also expect to see an increase over time in the use of natural sweeteners. Interestingly, despite the fact that natural sweeteners have the health halo of being plant-based, their uptake and use so far in NABs is limited (6%) [49]. A possible explanation for this observation could be that the process of identifying the right types and amounts of natural sweeteners to formulate the requisite sensory qualities of beverages is not a simple undertaking [52–55]. For instance, Stevia has been marketed as a promising renewable raw ingredient in the food industry for many years, but multiple studies have documented that its characteristic metallic, bitter aftertaste negatively influences consumer acceptance [54–56]. Another natural sweetener, monk fruit extract, is still a relatively new ingredient to the market with limited and inconclusive research examining its safety effects and sensory profile, to date as an additive [55]. While such challenges impose a hinderance, current direction shows that consumers are gravitating towards a clean, natural, and sustainable product label considering that society are becoming increasingly aware and interested in valuing their own health [35, 36]. We are keen to see how trends in natural sweeteners grow in beverages on the Singapore market. Moving forward, these natural sweeteners provide an alternative yet viable solution for the beverage industries to stay relevant to consumer preferences whilst meeting the requirements of health-based policies such as Nutri-Grade [15].

To the best of our knowledge, the present study is the first to be conducted which identifies and examines the types and proportion of sugar and/or sugar substitutes used in NABs sold on the Singapore market and available in the diet. It serves as a ‘point zero’ to track and evaluate the proportion of sugars in beverages as well as the amount of average sugar being displaced by sugar substitutes over time due to the upcoming implementation of the Nutri-Grade labelling system for all branded retail products.

It is acknowledged that the approach used in this study does not take into account sugar and/or sugar substitutes present in freshly prepared beverages sold over the counter such as Starbucks™ and McDonald’s® [11]. However, it was not the primary aim of the study to look at sugar intake per se, but rather, to examine pre-packaged beverages where labelling is mandatory for sugar. Over-the-counter products sold in Singapore do not require nutrient labelling nor any ingredient composition listing. In order to undertake a dietary exposure study of sugar and sweetener intake in Singapore, we intend to expand our categories to other beverages available through retail stores e.g., flavoured milk and milk alternatives, as well as investigate how to apply a similar data collection methodology to over-the-counter products. In addition, we are looking to expand our data collection across all product categories as part of the development of a national branded food database for Singapore and to investigate other labelling requirements. We view the collection of such market product data as an important tool to understand the dietary composition of Singaporeans, guide policy decisions and investigate topical research questions such as the one addressed in this paper.

## Conclusion

With continuous efforts by the government in promoting public health nutrition, it is expected that there will be a greater usage of sugar substitutes among NABs in response to the upcoming implementation of Nutri-Grade and ever-fluctuating consumers’ demands. Going forward, what effect(s) it has on the beverage composition will need to be further assessed. The comprehensive baseline data collected in this study provides a solid foundation on which to assess future changes in product formulae and the impact of the Nutri-Grade system on the composition of beverages available on the Singapore market and in the Singapore diet.

## Supplementary Information

**Additional file 1: Appendix 1.** Category Definition – Non-Alcoholic Beverages (NABs).

## Data Availability

The data used and/or analysed during the current study are available from the corresponding author on reasonable request.

## Abbreviations

HCS: Healthier Choice Symbol
HPB: Health Promotion Board
NABs: Non-Alcoholic Beverages
NIP: Nutritional Information Panel
SSBs: Sugar Sweetened Beverages
T2DM: Type 2 Diabetes Mellitus
