# Correction to: Assessment of artificial and natural sweeteners present in packaged non-alcoholic beverages (NABs) sold on the Singapore market

**DOI:** 10.1186/s12889-021-12342-y

**Published:** 2022-05-14

**Authors:** Rebecca Tan, Sharon Chew, Xenia Cleanthous, Kimberley Anastasiou, Paige G. Brooker, Theresa Pham, Benjamin P. C. Smith

**Affiliations:** 1 Singapore Institute of Food and Biotechnology Innovation & Innovations in Food & Chemical Safety Programme, Agency for Science, Technology and Research, Singapore, 138671 Singapore; 2 Health & Biosecurity, The Commonwealth Scientific and Industrial Research Organisation, ACT, Canberra, 2601 Australia; 3 National Heart Foundation Australia, Docklands, VIC 3008 Australia; 4 Future Ready Food Safety Hub, C/O School of Chemical & Biomedical Engineering, Nanyang Technical University, Singapore, 637459 Singapore

**Correction to: BMC Public Health 21, 1866 (2021)**

**https://doi.org/10.1186/s12889-021-11924-0**

In the original publication [1] there was an incorrect funding acknowledgement. In this correction article the correct and incorrect funding acknowledgement are published. The original article has been updated.

**Incorrect funding**

- This study was funded under the Singapore-Australia Bilateral Program on Innovations in Food for Precision Health 2019.

**Correct funding**

- This study was funded under the Singapore-Australia Bilateral Program on Innovations in Food for Precision Health 2019. BPC Smith was supported by a National Research Foundation Singapore Whitespace grant (grant no. W20W3D0002) and Health and Biomedical Sciences Industry Alignment Fund Pre-positioning grant (H1801a0–014) administered by the Agency for Science, Technology & Research.
