# Supplementary material for: Assessment of artificial and natural sweeteners present in packaged non-alcoholic beverages (NABs) sold on the Singapore market
Source: BMC Public Health. 2021 Oct 16;21:1866. doi: 10.1186/s12889-021-11924-0 (PMC8520227; doi:10.1186/s12889-021-11924-0)
Supplement: Supplementary file 1 — Additional file 1: Appendix 1. Category Definition – Non-Alcoholic Beverages (NABs). [file 12889_2021_11924_MOESM1_ESM.docx]

**Appendix**

Category Definition – Non-Alcoholic Beverages (NABs)

(to be collected as SUGAR-SWEETENED BEVERAGES in the App)

| **Category to Classify in App:** | - **Sugar-sweetened beverages** | | |
| --- | --- | --- | --- |
| **Definition:** | - All non-dairy, non-alcoholic beverages with added sweeteners (e.g., sucrose (‘sugar’), fruit-juice concentrates) - All artificially sweetened or intense-sweetened varieties that may be marketed as e.g., ‘reduced sugar’, ‘sugar-free’, ‘no added sugar’, or ‘diet’ | | |
| **Inclusions:** | - Soft drinks – e.g., Coke, Lemonade, Fanta, including ‘diet varieties’, Tonic water (exclude Soda Water, Club Soda, etc.) - Sparkling flavoured water drinks (main ingredient is usually water, flavouring is a small component) - Vitamin waters, Flavoured waters (include fruit and other plant-based flavours) - Sports/isotonic drinks – e.g., Powerade, Gatorade, etc. (include powders that need reconstitution) - Energy drinks – e.g., V, Mother, Red Bull, etc. - Other non-alcoholic Asian-style drinks/beverages – e.g., Barley drinks, Longan & Red Date, bandung drinks (include drinks with ‘bandung’ in product names), bubble teas, teas and other drinks with pearls/bubbles/jelly, etc. - Coconut water, plain and flavoured (include drinks with ‘coconut’ in product names) (include plain and flavoured varieties) - Ready-to-drink (RTD) products with ‘tea’ in product names which could be marketed as iced teas or milk teas. They could be chilled or self-stable, sweetened and/or flavoured (sugar or artificially) with or without added milk (where milk is added as an ingredient and not the characterising ingredient). These products are generally positioned in the same aisle as RTD SSBs - Iced coffees (chilled or shelf stable), sweetened and/or flavoured with or without added milk (where milk is added as an ingredient and not the characterising ingredient). These products are generally positioned in the same aisle as RTD SSBs - Cordials, syrups, concentrates (not marketed as a fruit juice), mixers and powdered flavour sachets (need reconstitution before consumption) - Vinegar products (where positioned and marketed as ‘drinking vinegar’) – e.g., CJ Petitzel Fruity Fruit, etc. - Kombucha/non-dairy based probiotic drinks/non-dairy based probiotic fruit drinks | | |
| **Exclusions:** | - Fruit and vegetable juices and drinks (include those made from fruit juice concentrates; sparkling fruit drinks) – these fit into the **‘Fruit Juice’** and **‘Vegetable Juice’** categories - Tea bags, instant coffee, coffee capsules and pods – these fit into the **‘Tea and Coffee**’ category - Plain and flavoured milk-based drinks made from dairy alternatives with ‘milk’ in the product name – these fit into the **‘Milk and Milk Alternatives’** category - Dairy-based iced tea and coffee, e.g., Nescafé Milk Coffee, where dairy/dairy-alternative is the main/characterising ingredient – these fit into the **‘Milk and Milk Alternatives’** category - Drinking yoghurts – these fit into the **‘Yoghurt and Dairy Desserts**’ category - Plain water without any added flavourings and compounds – e.g., still, soda, sparkling – these fit into the **‘Water’** category - Vinegars positioned within the salad dressing section and are marketed as standard vinegar (not to be consumed as a beverage) – these fit into the **‘Salad Dressings and Mayonnaise’ c**ategory - Baby/infant foods and formula – these will not be collected - Alcoholic beverages – these will not be collected | | |
| **Packaging:** | - Cans, bottles, cartons (e.g., Tetra Pak), sachets, in singular and multipacks (of various volumes e.g., 320 mL can, 1.5L bottle) | | |
| **Typical Positions in Supermarkets:** | - Chilled section (fridge, multi-deck open chillers, at point of sale) – mostly single serve bottles, cans - Majority of products found in the shelf-stable drinks aisle (grouped together for convenient access to various beverages) - Cordials can be positioned near fruit juices (depending on the supermarket and store layout) - Promotional items may be located at highly visible areas of the supermarket (e.g., front of store, area before cashier check-out counters, at both ends of supermarket aisles) and are labelled with larger discount display cards | | |
| **NON-ALCOHOLIC BEVERAGE PRODUCTS DURING ONLINE STORE SCOPING** | | | |
| **Manufacturer/Retailer** | | **Brand** | **Country of Origin** |
| Allswell Asian Beverages | | Allswell | Taiwan |
| Aqua Maestro, Inc | | San Benedetto | Italy |
| ASF Food & Beverage (M) | | Asia Farm, Giant | Malaysia |
| Asiatic Agro Industry Co. | | COCOmax | Thailand |
| Bragg Live Food Products, Inc. | | BRAGG | USA |
| BUNDABERG BREWED DRINKS | | BUNDABERG | Australia |
| CJ CheilJedang Corp. | | CJ Petitzel | South Korea |
| The Coca-Cola Company [various countries]. May also be displayed on pack as COCA-COLA AMATIL, Coca-Cola Bottlers, Coca-Cola Refreshments Malaysia | | AQUARIUS, A&W, AUTHENTIC TEA HOUSE, Coca-Cola, Coca-Cola Signature Mixers, FANTA, Glaceau, Heaven and Earth, Honest Tea, Minute Maid, Monster Energy, Schweppes, Sprite, Vitaminwater, ZICO | Hong Kong, Malaysia, Singapore, Thailand, UK, USA |
| Etika | | 7 Up, EVERVESS, GATORADE, MiRiNDA, Mountain Dew, MUG, Pepsi | Malaysia |
| F&N Beverages Manufacturing | | F&N, F&N ICE MOUNTAIN, F&N SEASONS |  |
| F&N Foods | | 100PLUS, COCO Life, F&N, F&N ICE MOUNTAIN, F&N NutriWell, F&N Seasons | Malaysia, Philippines, Singapore |
| Fevertree | | FEVER-TREE | UK |
| Healthy Food Brands | | Switchle |  |
| JiaJia (JJ) Drinks Manufacturing | | JJ | Singapore |
| KEKO Marketing (M) | | Glinter | Malaysia |
| Krating Daeng | | Red Bull | Thailand |
| Life | | Life | Japan, Taiwan |
| Malaysia Milk | | MARIGOLD | Malaysia |
| Mamami | | Mamami | Thailand |
| Mondelēz International | | TANG – Orange Instant Drink Mix | Bahrain |
| NA (unavailable – check in store) | | Du Siang | Taiwan |
| Naspac Marketing | | DRAGON COIN BRAND | Malaysia |
| Nestlé, SANPELLEGRINO S.p.A. | | SANPELLEGRINO | Italy |
| Nestlé Waters | | Perrier | France |
| Ng Nam Bee Marketing | | DAY’O Cordial | Malaysia |
| Otsuka Pharmaceuticals | | Pocari Sweat | Japan |
| PepsiCo [various countries] | | 7 UP, Gatorade, Mirinda, Mountain Dew, Pepsi | Malaysia, Singapore, USA |
| POKKA Corporation | | POKKA, Sparklin’ | Malaysia, Singapore |
| Private Label – Cold Storage | | CASINO, WAITROSE, WAITROSE Essential | NA (varies) |
| Private Label – FairPrice | | FairPrice | Malaysia, Thailand |
| Private Label – Giant | | Giant | NA (varies) |
| Private Label – 7-Eleven | | 7-Premium | Japan |
| PT. Amerta Indah Otsuka | | POCARI SWEAT | Indonesia |
| Red Bull Australia | | Red Bull | Australia |
| Remedy Drinks | | Remedy |  |
| Sheng Sheng F&B Industries | | Ice Cool | Thailand |
| Shih-Chuan Excellence Food Co. | | Shih-Chuan | Taiwan |
| Starbucks Corporation | | Starbucks | USA |
| Suma Co-operative | | Suma | UK |
| Suntory Beverage & Food Malaysia | | Orangina, Ribena, Suntory | France, Malaysia, Taiwan |
| Tesco Stores | | TESCO | Thailand |
| T.G. KIAT & CO | | ROSE BRAND – Rose Syrup | Singapore, UK |
| UNIVERSAL FOOD PLC. | | UFC | Thailand |
| Vitasoy International Holdings | | ViTa | Hong Kong, Malaysia |
| Windmill Organics | | BIONA Organic | UK |
| Yeo Hiap Seng, YHS | | KICKAPOO, Yeo’s | Malaysia, Singapore, Taiwan, Thailand |
| Yit Hong | | YIFON | China |

**ADDITIONAL NON-ALCOHOLIC BEVERAGE PRODUCTS DURING OFFICIAL IN-STORE DATA COLLECTION PROCESS**

| **Manufacturer/Retailer** | **Brand** | **Country of Origin** |
| --- | --- | --- |
| A.G.BARR | IRN BRU | UK |
| ACE CANNING CORPORATION | DRINHO | Malaysia |
| Acetificio Marcello de Nigris SRL | DE NEGRIS | Italy |
| Alce Nero Asia | Alce Nero |  |
| Allswell Trading | ALLSWELL | Singapore |
| AriZona Europe Coöperatief U.A. | AriZona | Netherlands |
| ASAHI BEVERAGES | Schweppes, SOLO | Australia |
| BARKATH CO-RO MANUFACTURING | SUNQUICK | Malaysia |
| Ben Food (S) | Orchard Fresh | Belgium, Korea |
| BICKFORD’S AUSTRALIA | BICKFORD’S | Australia |
| Bod Conscious Enterprises | bod |  |
| BREWGURU CO. | BrewGuru | Korea |
| BTC LLP | EIGHTY 8 | UK |
| Bunderim Group | Bunderim Ginger | Australia |
| CAWSTON PRESS | CAWSTON PRESS | UK |
| Chen Jiah Juang Natural Agriculture Co. | Chen Jiah Juang | Taiwan |
| Chia Khim Lee Food Industries | Gold Cow, Sagiko | Singapore, Vietnam |
| Citrus Marketing | Harvest | Singapore |
| CJ CheilJedang Corp. | CJ Petitzel | Korea |
| COMPAGNIA ALIMENTARE ITALIANA | FRATELLI MANTOVA | Italy |
| DFI Brands | Meadows | Malaysia |
| FAMOUS HOUSE FOOD INDUSTRIAL CORP. | FAMOUS HOUSE |  |
| Field Catering & Supplies | POLAR, SINGA, Sportade | Malaysia, Singapore, Taiwan |
| Gaya Farm | GAYA FARM | Korea |
| General Beverage Co. | if LOCAL SENSATION | Thailand |
| Genki Forest Beverage Co. | Genki Forest | China |
| Gerolsteiner Brunnen | GEROLSTEINER | Germany |
| GOURMET BREWERY | HAUS BREW | Singapore |
| Gryphon Tea Company | GRYPHON TEA CO. | Malaysia |
| GUANGZHOU PRESIDENT ENTERPRISES CORP. | Unif | China |
| HeySong Corporation | HeySong Cherico, Lipton | Taiwan |
| Honest Tea Inc. | Honest T | USA |
| Honey Corporation of Australia | Barnes naturals | Australia |
| Indoguna Singapore | CHA | Taiwan |
| ITO EN | ITO EN | Thailand |
| Itoh Kanpo Pharmaceutical Co. | ITOH | Japan |
| JUST PICKED COCOWATER | just picked COCO | Thailand |
| Keurig Dr Pepper | Snapple | USA |
| KIRIN | KIRIN’S World Kitchen | Japan |
| Kirin Beverage Co. | KIRIN |  |
| Kirin Holdings Company |  |  |
| KUANG CHUAN DAIRY CO. | Kuang Chuan | Taiwan |
| Li Tat Food & Beverages Manufacturing (M) | ICE Cool | Malaysia |
| LINACO MANUFACTURING (M) | Rasaku |  |
| Living Essentials LLC | 5-hour ENERGY | USA |
| Lotte Chilsung Beverage Co. | LOTTE | Korea |
| MALAYSIA POKKA Corporation (S) | POKKA | Malaysia |
| Margaret River Kombucha Co. | rok Kombucha | Australia |
| MAXMASTER INDUSTRY | Giant | Malaysia |
| MAYA CORP. | Asina |  |
| Min Hiang Food | FIRST BREW | Singapore |
| MONSTER ENERGY COMPANY | MONSTER | USA |
| N&N | N&N | Singapore |
| N.W.S., Nestlé Waters | Perrier | France |
| Namyang Dairy | Chupa Chups | Korea |
| Nongfu Spring | Nongfu Spring | China |
| OCEANIC BEVERAGES CO. | OCEANIC BEVERAGES | Taiwan |
| P.C.I. Professional Canning Industries, Sheng Sheng F&B | ICE Cool | Malaysia,  Singapore,  Thailand |
| Pai Chia Chen Brewery & Foods Co. | Pai Chia Chen | Taiwan |
| Pan Industry Fareast Group Co. | Golden Pan | Thailand |
| Pepsi Lipton | Lipton | UK |
| POKKA | POKKA | Malaysia, Singapore |
| POLAR | POLAR | Taiwan |
| PT Djojonegoro C-1000 | YOU-C1000 | Indonesia |
| PT. COCA-COLA BOTTLING | Coca-Cola, Sprite |  |
| PT. Sinar Sosro | SOSRO |  |
| PURE BIO* PRODUCTS | PURE BIO | Austria |
| Red Bull Asia FZE, T.C. Pharmaceutical Industries Co. | Red Bull | Austria, Thailand |
| Ribena Suntory | Lucozade | UK |
| S&W FINE FOODS INTERNATIONAL | S&W | USA |
| Sappe Public Company | MOGU MOGU | Thailand |
| Siam Coconut | COCOLOCO |  |
| SLADES BEVERAGES | ANGUS O’NEILS’S | Australia |
| SOULFRESH GLOBAL | LO BROS |  |
| Suntory Beverage & Food Malaysia | Good Mood | Malaysia |
| Taiwan Morinaga Co. | Weider | Taiwan |
| THE GATORADE CO. | GATORADE | Malaysia |
| TIPCO F&B | Tipco | Thailand |
| TRIKO FOODS CO. | SHENG HSIANG JEN | Taiwan |
| U-GLOBE INTERTRADE CO. | Uglobe | Thailand |
| UNI-PRESIDENT ENTERPRISES CORP. | Uni-President | Taiwan |
| Waitrose | Essential Waitrose, Waitrose | UK |
| WAN TAI FENG FOOD CO. | MIZUMI | Taiwan |
| Wong Coco | WONG COCO | Singapore, Thailand |
| Woongjin Foods Co. | Woongjin | Korea |
| Xiang Piao Piao Co. | Xiang Piao Piao | China |
| Xinzheng Huakaixing Food Co. | White Rabbit |  |
| Yocha | Yocha | Singapore |
